# Durable response to mTOR inhibition in a patient with relapsing papillary tumor of the pineal region

**DOI:** 10.1093/neuonc/noy153

**Published:** 2018-10-05

**Authors:** Mattias Belting, Anna Bång-Rudenstam, Elisabet Englund, Torsten Pietsch

**Affiliations:** 1 Department of Clinical Sciences, Lund University, Lund, Sweden; 2 Department of Hematology, Oncology, and Radiophysics, Skåne University Hospital, Lund, Sweden; 3 Institute of Neuropathology, DGNN Brain Tumor Reference Center, University of Bonn, Germany

Papillary tumor of the pineal region (PTPR) was first described as a distinct tumor entity by Jouvet et al in 2003^1^ and was eventually included in the 2007 World Health Organization (WHO) classification of CNS tumors as a rare neuroepithelial tumor characterized by papillary architecture and epithelial cytology.^2^ PTPR mostly occurs in younger adults and shows frequent local recurrence after surgery, which corresponds to WHO grade II or III.^2^ So far, there is no established oncological treatment of these patients. We previously described that loss of chromosome 10 and phosphatase and tensin homolog (PTEN), and associated pathological activation of the Akt/mammalian target of rapamycin (mTOR) pathway, is a hallmark of PTPR.^3^ Here, we for the first time report a patient case with relapsing and rapidly progressing PTPR that showed loss of chromosome 10 and PTEN, phosphatidylinositol-3 kinase (PI3K)/Akt/mTOR hyperactivity, and a dramatic and durable response to mTOR inhibition with everolimus.

The patient is a 28-year-old male with PTPR diagnosed in 2011 with characteristic immunophenotype following subtotal resection. The DNA methylation-based classification revealed a confident classifier score for the methylation group “PTPR subtype 2.” These results were in line with the histological and immunohistochemical data and were consistent with a relatively poor prognosis, with a reported mean progression-free survival of 43 months in this subgroup.^4^,^5^ More importantly, a copy number profile extracted from the DNA methylation profile demonstrated several chromosomal aberrations, most notably loss of chromosome 10 (Fig. 1A). Chromosome 10 harbors the *PTEN* gene, which acts as a major tumor suppressor through its negative regulation of the PI3K/Akt/mTOR pathway. Accordingly, immunohistochemical examination revealed lost expression of PTEN in tumor cells as well as strong staining for phosphorylated Akt (Fig. 1A), which is consistent with a pathological activation of the pathway.

**Fig. 1:**
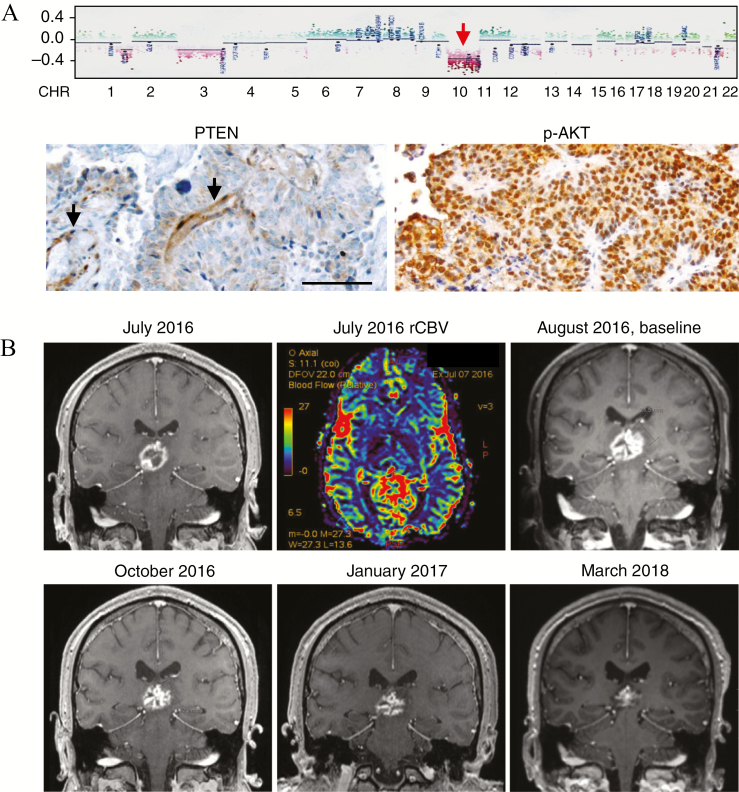
Loss of chromosome 10 and PTEN, hyperactivation of the PI3K/Akt/mTOR pathway, and response to treatment with everolimus. (A) DNA methylation array data revealed loss of chromosome 10 (red arrow), which harbors the *PTEN* gene (upper panel). The PTPR specimen shows negative immunohistochemical staining for PTEN in tumor cells (lower left). Arrows indicate positively stained vascular cells as internal positive control. The majority of tumor cells were strongly positive for p-Akt (lower right). Scale bar, 100 µm. (B) Gadolinium enhanced, T1-weighted coronal MRI demonstrating tumor progression and enhanced relative cerebral blood volume (rCBV) at the indicated dates. Everolimus treatment was initiated in August 2016. Serial MRI after approximately 2, 5, and 19 months of everolimus treatment demonstrated a marked radiological response.

The patient showed recurrent relapses on radiosurgical treatment as well as radiochemotherapy (30 × 2 Gy and temozolomide). In July 2016, MRI showed increased tumor volume, intensified contrast enhancement, and enhanced relative cerebral blood volume (rCBV) consistent with recurrent disease (Fig. 1B). Everolimus treatment at standard dosage of 10 mg/d was initiated in August 2016 in a situation where the patient’s clinical status continued to deteriorate with progressive impairment of eye movement and disturbed sensitivity and coordination and disabling weakness of the upper left limb. Follow-up MRI showed successive regression of contrast enhanced tumor. The patient displayed no side effects, and it was decided to increase dosage to 12.5 mg/d; however, the patient then developed acneiform dermatitis (ie, cutaneous eruptions resembling acne with pustules and comedones) on the face, neck, and scalp. These symptoms were efficiently alleviated by tetracycline treatment and dose-reduction of everolimus that was given at 5 mg/d from May 2017 and is still ongoing. During the entire treatment period, the patient received no other additional therapy.

Nineteen months after initiation of everolimus treatment, the volume of contrast enhanced tumor in the pineal region had decreased by ~75% (from 30 × 24 × 22 mm to 19 × 16 × 14 mm), and contrast enhanced areas in the thalamus, mesencephalon, and splenium corpus callosum showed partial to complete regression (Fig. 1B). This was associated with a remarkable improvement of the patient’s performance status and quality of life, including normalized sensory function and substantial recovery of motor function in the left arm and hand.

This case indicates that PTPR—even after repeated relapses—depends on pathological mTOR activity and that mTOR inhibition represents a feasible option for targeted treatment in these patients. Based on the biological rationale and the well-documented response of the presented case, we conclude that everolimus should be considered as first-line treatment in patients with recurrent PTPR.

## Funding

The study received grant support from the Skåne University Hospital donation funds, the Medical Faculty, Lund University, governmental funding of clinical research within the national health services (ALF) (to M.B.), and the German Childhood Cancer Foundation (Deutsche Kinderkrebsstiftung) (to T.P.)

## 

**Conflict of interest statement**. The authors have no conflicts of interest to declare.

## References

[1] Jouvet A, Fauchon F, Liberski P, et al Papillary tumor of the pineal region. Am J Surg Pathol. 2003;27(4):505–512. 12657936 10.1097/00000478-200304000-00011

[2] Louis DN, Ohgaki H, Wiestler OD, Cavenee WK. WHO Classification of Tumours of the Central Nervous System, revised 4th ed. Lyon: IARC; 2016.

[3] Goschzik T, Gessi M, Denkhaus D, Pietsch T PTEN mutations and activation of the PI3K/Akt/mTOR signaling pathway in papillary tumors of the pineal region. J Neuropathol Exp Neurol. 2014;73(8):747–751. 25003235 10.1097/NEN.0000000000000093

[4] Fèvre-Montange M, Hasselblatt M, Figarella-Branger D, et al Prognosis and histopathologic features in papillary tumors of the pineal region: a retrospective multicenter study of 31 cases. J Neuropathol Exp Neurol. 2006;65(10):1004–1011. 17021405 10.1097/01.jnen.0000240462.80263.13

[5] Heim S, Sill M, Jones DT, et al Papillary tumor of the pineal region: a distinct molecular entity. Brain Pathol. 2016;26(2):199–205. 26113311 10.1111/bpa.12282 PMC8029206

